# Heritability of alpha and sensorimotor network changes in temporal lobe epilepsy

**DOI:** 10.1002/acn3.51032

**Published:** 2020-04-25

**Authors:** Siti N. Yaakub, Chayanin Tangwiriyasakul, Eugenio Abela, Michalis Koutroumanidis, Robert D. C. Elwes, Gareth J. Barker, Mark P. Richardson

**Affiliations:** ^1^ Department of Basic & Clinical Neuroscience Institute of Psychiatry King’s College London Psychology & Neuroscience London UK; ^2^ School of Biomedical Engineering & Imaging Sciences King’s College London & Guy’s and St Thomas’ PET Centre King’s College London London UK; ^3^ Department of Clinical Neurophysiology King’s College Hospital NHS Foundation Trust London UK; ^4^ Department of Clinical Neurophysiology and Epilepsies Guy’s and St. Thomas’ NHS Foundation Trust St. Thomas’ Hospital London UK; ^5^ Department of Neuroimaging Institute of Psychiatry, Psychology & Neuroscience King’s College London London UK

## Abstract

**Objective:**

Electroencephalography (EEG) features in the alpha band have been shown to differ between people with epilepsy and healthy controls. Here, in a group of patients with mesial temporal lobe epilepsy (mTLE), we seek to confirm these EEG features, and using simultaneous functional magnetic resonance imaging, we investigate whether brain networks related to the alpha rhythm differ between patients and healthy controls. Additionally, we investigate whether alpha abnormalities are found as an inherited endophenotype in asymptomatic relatives.

**Methods:**

We acquired scalp EEG and simultaneous EEG and functional magnetic resonance imaging in 24 unrelated patients with unilateral mTLE, 23 asymptomatic first‐degree relatives of patients with mTLE, and 32 healthy controls. We compared peak alpha power and frequency from electroencephalographic data in patients and relatives to healthy controls. We identified brain networks associated with alpha oscillations and compared these networks in patients and relatives to healthy controls.

**Results:**

Patients had significantly reduced peak alpha frequency (PAF) across all parietal and occipital electrodes. Asymptomatic relatives also had significantly reduced PAF over 14 of 17 parietal and occipital electrodes. Both patients and asymptomatic relatives showed a combination of increased activation and a failure of deactivation in relation to alpha oscillations compared to healthy controls in the sensorimotor network.

**Interpretation:**

Genetic factors may contribute to the shift in PAF and alterations in brain networks related to alpha oscillations. These may not entirely be a consequence of anti‐epileptic drugs, seizures or hippocampal sclerosis and deserve further investigation as mechanistic contributors to mTLE.

## Introduction

Mesial temporal lobe epilepsy (mTLE) is the most common type of medically refractory focal epilepsy in adults. Most adults with mTLE present with hippocampal atrophy or hippocampal sclerosis (HS), which may be amenable to surgical treatment.^1^ Abnormalities in patients with HS extend beyond the hippocampus and are thought to involve a network of regions including temporal, thalamic and limbic regions^2^; evidence for whether abnormalities precede seizure onset or are a result of long‐term seizures is scarce and mixed.^3^, ^4^ While mTLE is generally thought of as an acquired disorder, there is emerging evidence for a genetic link in sporadic mTLE,^5^, ^6^ and studies have found alterations in structural brain morphology in asymptomatic relatives of patients with HS.^7^, ^8^ This structural alteration in asymptomatic relatives suggests an inherited trait that precedes seizure onset.^9^

Alpha is one of the main background electroencephalography (EEG) physiological rhythms observed primarily over the bilateral posterior (occipital) areas when subjects are awake and relaxed, and their eyes are closed. Its frequency, from late childhood through adulthood is within the 8–13 Hz range. Alpha activity is typically attenuated (or blocked) by both visual and non‐visual stimuli and mental tasks.^10^ Recent studies have shown evidence for the active involvement of alpha activity in cognitive processes,^11^, ^12^, ^13^ and an emerging theory is that the alpha‐rhythm governs neural excitability through top‐down modulated cognitive control networks.^14^ The neural substrates underpinning alpha activity are still not well understood but are thought to be thalamo‐cortical in origin,^15^ based on animal models^16^, ^17^, ^18^, ^19^ and neuroimaging studies in humans.^20^, ^21^, ^22^, ^23^, ^24^

It is known that there are alterations in alpha activity in patients with epilepsy, but these alterations are rarely reported or described.^25^ While it is known that alpha activity is influenced by, among other things, anti‐epileptic drugs,^26^, ^27^ reductions in peak alpha frequency (PAF) have been shown to be epilepsy‐specific^25^ and to distinguish between focal and generalized epilepsy^28^ and changes in both frequency and power of alpha activity are related to the severity and type of seizures after accounting for anti‐epileptic drug load.^28^, ^29^

Alpha power and peak frequency have been shown to be highly reproducible within individuals,^30^, ^31^ and highly heritable.^32^, ^33^ There exists a large amount of inter‐individual variation related to age, memory and cognition, and neurological conditions,^34^, ^35^, ^36^, ^37^, ^38^ which suggests that alpha activity measures may be too unspecific to be considered biomarkers or endophenotypes.^14^ However, there is some evidence for a brain network endophenotype for idiopathic generalized epilepsy derived from the “low alpha” frequency band.^39^ It is so far unknown whether there may be functional or structural network alterations related to alpha activity in relatives of patients with mTLE.

The present study seeks to characterize EEG alpha band power and peak frequency in patients with mTLE and asymptomatic relatives compared to healthy controls and investigate whether the functional brain networks related to alpha oscillations differ in patients with mTLE and asymptomatic relatives compared to healthy controls.

## Materials and Methods

### Participants

The study was performed at the National Institute for Health Research/Wellcome Trust King’s Clinical Research Facility at King’s College Hospital, London, United Kingdom. All experimental procedures were reviewed and approved by the London – Bromley National Research Ethics Service. Written informed consent was obtained from each participant after all procedures were fully explained.

Twenty‐four unrelated patients with mTLE were recruited from outpatient epilepsy and neurology clinics in hospitals in south London. The diagnosis of mTLE was made on the basis of clinical evaluation including history, seizure semiology, scalp EEG, and conventional clinical MRI reported by experienced neuroradiologists. Patients who had other pathologies, for example, malformations of cortical development or tumors, who had undergone surgical resection of the affected temporal lobe, or who had recent invasive brain investigations (including depth electrode recordings) were excluded from the study.

Twenty‐three asymptomatic first‐degree relatives were recruited either through patients included in the study or through patients who had a diagnosis of mTLE but were themselves excluded from the study due to a history of surgical resection or recent invasive brain investigations. (Note that recruiting in this way means that while we refer to them as “relatives”, some members of this group have no patient to whom they are related in the patient group). Thorough clinical interview of these relatives revealed no current or previous diagnosis of neurological disorders, and no history of symptoms or clinical events suggestive of epileptic seizures. Scalp EEG, carried out as part of the study, showed no epileptiform discharges in any relative.

Thirty‐two healthy control participants with no current or previously diagnosed personal or family history of neurological disorders were recruited for comparison (Table 1A).

**Table 1:** Demographic information for all participants and clinical information for patients.

	Patients	Relatives	Controls
(A) Participants with EEG data only			
Number	24	23	32
Age (years)	40.2 ± 11.9	36.7 ± 13.0	36.9 ± 10.8
Sex (male/female)	13/11	10/13	16/16
mTLE side^1^ (right/left)	10/14	9/14	–
Epilepsy onset age (years)	21.8 ± 9.9	–	–
Duration of epilepsy (years)	18.0 ± 14.0	–	–
Seizure frequency (/month)^2^	5.6 ± 6.3	–	–
(B) Participants with simultaneous EEG‐fMRI data			
Number	22	18	25
Age (years)	39.3 ± 12.7	35.9 ± 13.3	34.8 ± 7.9
Sex (male/female)	11/11	8/10	13/12
mTLE side^1^ (right/left)	9/13	8/10	–
Epilepsy onset age (years)	23.1 ± 9.1	–	–
Duration of epilepsy (years)	15.6 ± 13.7	–	–
Seizure frequency (/month)^2^	5.8 ± 6.6	–	–

Data are means ± standard deviations. EEG, electroencephalography; mTLE, mesial temporal lobe epilepsy; fMRI, functional MRI.

^1^ Refers to side of seizure onset in patients group, and of the proband in relatives group.

^2^ Data unavailable for one patient.

Full clinical information for patients is given in Table S1 and details of relatives and clinical information of their associated probands given in Table S2.

### Data acquisition

#### Study design

Participants had a 20‐min EEG recording outside the MRI scanner, a high‐resolution structural T1‐weighted MRI scan and a 10‐min simultaneous EEG and functional MRI (fMRI) recording in the scanner. During EEG and fMRI recordings, participants were instructed to stay awake and relax with their eyes closed.

To increase power to detect pathological differences, imaging and EEG data from patients with right‐sided mTLE (*n* = 7; 38.9%) and relatives of patients with right‐sided mTLE (*n* = 5; 41.7%) were left‐to‐right flipped so that the ipsilateral side is on the left. All changes were considered as ipsilateral or contralateral to the pathological hippocampus in patients. For consistency, a similar proportion of healthy control data (*n* = 12; 38.7%) was randomly chosen to be side flipped.

Not all participants completed the simultaneous EEG and fMRI investigations due to reasons including claustrophobia and equipment problems. The subset of participants with complete data is described in Table 1B.

#### EEG data

EEG data were recorded using a 64‐channel MRI‐compatible EEG system (Brain Products GmbH, Munich, Germany). All participants were fitted with a BrainCap MR EEG cap with 63 Ag/AgCl electrodes arranged according to the extended international 10–20 system with the reference electrode placed between Fz and Cz and the ground between Fz and Fpz. The electrocardiogram (ECG) was recorded at a sampling frequency of 5 kHz using the BrainVision Recorder software (Brain Products). EEG recordings outside the scanner were performed in an electrically shielded Faraday cage room.

#### MRI data

MRI was performed on a General Electric 3T MR750 scanner (GE Healthcare Systems, Chicago, IL) using the body coil for radiofrequency transmission and a 12‐channel head coil for signal reception. Resting‐state fMRI data were acquired using a gradient‐echo echo‐planar imaging sequence in a plane parallel to the AC‐PC line, 2160 msec repetition time (TR), 25 msec echo time, 75° flip angle, 36 interleaved slices of 64 × 64 matrix size, giving a 211 × 211 mm field of view with a voxel size of 3.3 × 3.3 × 3.3 mm. Simultaneous EEG was recorded during the fMRI scan at a sampling frequency of 5 kHz with the SyncBox device (Brain Products) used to synchronize EEG and fMRI acquisition. A three‐dimensional inversion recovery‐prepared spoiled gradient‐echo image was acquired in the sagittal plane with 270 mm field of view, 256 × 256 matrix (resulting in an in‐plane voxel size of 1.05 × 1.05 mm), 196 sagittal slices, 1.2 mm slice thickness, 7.312 msec TR, 400 msec inversion time, 3.016 msec echo time, and 11° excitation flip angle.

### Data analysis

#### EEG power spectral analysis

EEG data acquired outside the scanner was used for the power spectral analysis conducted in MATLAB (R2015b, The MathWorks Inc., Natick, MA, 2015) using tools from the FieldTrip EEG software toolbox.^40^ In previous work, we have shown that the choice of a segment does not affect the analysis.^28^ We conducted the analysis on the first 5 min of EEG recording to exclude any possible confounding effect of the choice and length of segments. Data were re‐referenced to the average of all channels except the Fp1, Fp2 and ECG electrodes, and de‐trended. Segments were visually inspected for artifacts and the presence of interictal discharges in patients.

Data were bandpass filtered between 0.5 and 70 Hz and the power spectral density for each participant’s segment was estimated using Welch’s method with a window length of 4 sec and 50% overlap, giving a frequency resolution of 0.25 Hz. The relative power at each frequency resolution point was computed as a fraction of the total power between 0.5 and 70 Hz. Data were band‐passed within the alpha frequency band (6–13 Hz) with the lower boundary of the alpha band modified to include “low alpha” frequencies,^41^ since previous work has shown alterations in the low alpha band in epilepsy.^28^, ^39^ The peak power (maximum power) and peak frequency (frequency at which the maximum power occurs) were computed for each subject. Statistical group comparisons of peak power and peak frequency were restricted to parietal and occipital channels, where the alpha rhythm is most prominently expressed. We used one‐tailed two‐sample *t*‐tests (with the hypothesis that patients and relatives would show reduced alpha power and frequency compared to healthy controls) and controlled the false discovery rate (FDR; over 17 parietal and occipital channels) using the Benjamini‐Yekutieli procedure with *α* = 0.05.

We performed three sub‐group analyses to investigate the effects of medication, seizure control and relatedness. Since carbamazepine is known to cause slowing of the alpha frequency, we split the patient group into patients taking carbamazepine (*n* = 8) and patients who were not (*n* = 16). To examine the effect of seizure control, we split the patient group into patients with good (*n* = 4) and poor (*n* = 20) seizure control (with the threshold for poor seizure control at ≥4 seizures/year, as defined in Ref. ^28^). We had seven pairs of related patients and relatives in this analysis. In the final sub‐group analysis, to exclude any confounding effects of relatedness, we excluded four patients and three relatives so that all patients and relatives remaining in the analysis were unrelated. In each of these sub‐group analyses, peak power and frequency in the alpha band were compared to healthy controls as described above.

#### Simultaneous EEG‐fMRI

EEG data recorded in the scanner were pre‐processed using BrainVision Analyzer (version 2.0, Brain Products) to remove MR gradient and ballistocardiogram artifacts from the simultaneous EEG‐fMRI data. A sliding average template of MRI scanner artifacts using the average of 21 TR intervals identified by the gradient onset markers was subtracted from the EEG signal to correct for MR gradient artifacts. Data were downsampled to 250 Hz. The peaks of the R‐waves were identified from the ECG signal in a semi‐automated manner using a template pulse wave, and subsequently visually checked and adjusted. Ballistocardiogram artifacts were corrected by subtracting a sliding average template for the R‐waves from the EEG.

The alpha power time‐series was extracted from the pre‐processed in‐scanner EEG data using MATLAB. EEG data were averaged over the three occipital electrodes (O1, O2, and Oz) and a short‐time Fourier transform was applied to compute the spectrogram using windows equal to the fMRI data sampling TR of 2.16 sec with no overlap. To account for intra‐subject variation of PAF, the alpha power time series was computed as the mean alpha power over a narrow band of ±2 Hz around the PAF for each subject. Outliers in the mean alpha power time series were identified as data points where the amplitude exceeded the standard boxplot outlier definition, Q3 + 1.5 × IQR, and replaced by linearly interpolated values. Each alpha power time series was then truncated to exclude the first 5 and last 5 TR of data to exclude end effects from average template correction and scaled between 0 and 1.

FMRI data were pre‐processed using FSL’s FEAT software (v6.0).^42^ FMRI data pre‐processing involved motion correction, spatial smoothing with a 6mm full width at half maximum Gaussian filter, and bandpass filtering between 0.01 and 0.12 Hz. Each subject’s native space data were co‐registered to the Montreal Neurological Institute (MNI) standard space image by way of a linear transform to the subject’s high‐resolution T1‐weighted image and then a non‐linear registration to standard space. To model and exclude the effects of noise within the data, several nuisance regressors were used as variables of non‐interest in the subsequent general linear model analysis. These were the average signal each from the white matter and cerebrospinal fluid, and the six motion parameters.

For each subject, a voxel‐wise whole‐brain general linear model analysis was implemented with FEAT with the alpha power time series as the variable of interest and the nuisance regressors as variables of non‐interest. The alpha power time series was convolved with the canonical double‐gamma hemodynamic response function to take into account the delay associated with the blood‐oxygen‐level‐dependent fMRI (BOLD‐fMRI) response. Group analyses were performed in FEAT with a mixed‐effects analysis of covariance model with age and sex as covariates. Contrasts for the main effect of group and group differences between healthy controls and each of the patient and relative groups were set up and significant effects were identified with a cluster threshold of *P* < 0.05, FWE‐corrected.

We used the normalized alpha power estimated from the O1, O2 and Oz electrodes as a measure of vigilance during the simultaneous EEG‐fMRI recording.^43^ For each subject, we estimated the normalized alpha power averaged across the three occipital electrodes using 10‐sec sliding windows across the in‐scanner EEG data (after discarding the first and last 5 TR as above). We then estimated the slope of the normalized alpha power for each subject and compared this between groups using a one‐way analysis of variance.

## Results

The participant groups were not significantly different in age or gender for any of the datasets when assessed using one‐way analyses of variance and χ^2^ tests.

### Alterations in the EEG power spectrum

**Figure 1:**
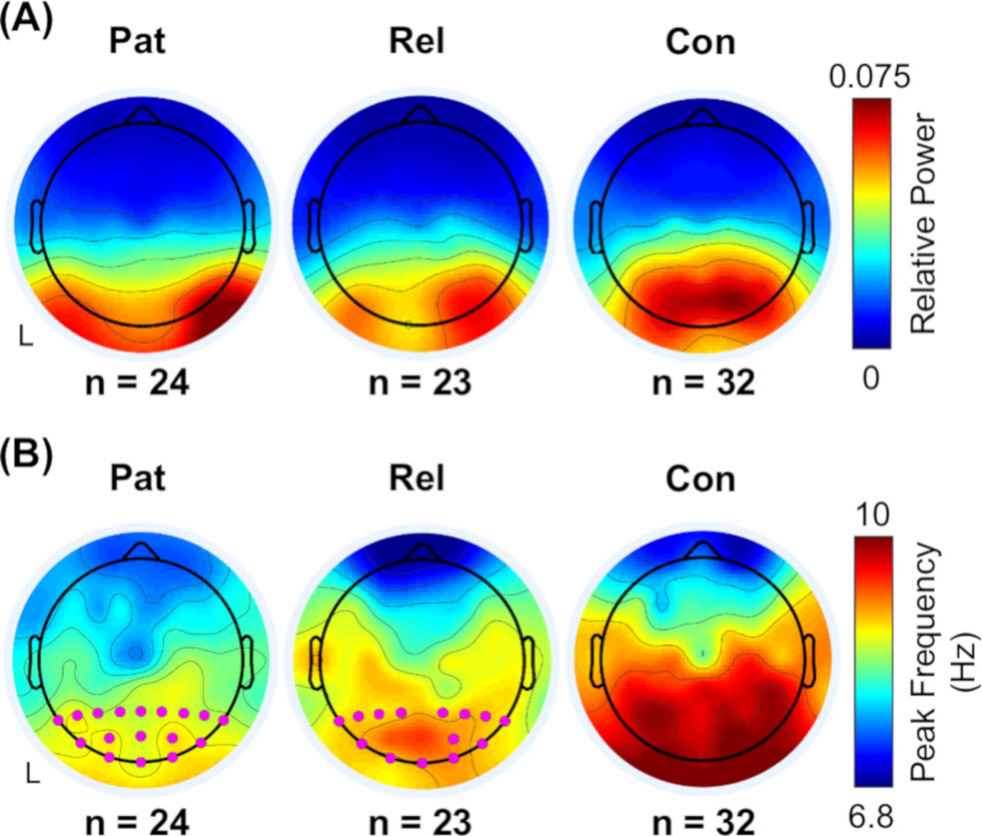
EEG topographical plots for the alpha band. Group‐averaged EEG topographical plots of (A) peak power and (B) peak frequency in the alpha frequency band. In the patients’ and relatives’ plots, channels that show a significant group difference from healthy controls are indicated by pink dots (*P* < 0.05, FDR‐corrected across parietal and occipital channels only). “L” indicates the left or ipsilateral side. Pat, patients with mTLE; Rel, asymptomatic relatives; Con, healthy controls; EEG, electroencephalography; FDR, false discovery rate; mTLE, mesial temporal lobe epilepsy.

Peak power and peak frequency within the alpha frequency band were averaged across group, and patients with mTLE and asymptomatic relatives were compared to healthy controls. No significant differences in peak power in either patients or relatives from controls were present (Fig. 1A). PAF was significantly reduced in patients with mTLE compared to healthy controls across all parietal and occipital channels (*P* < 0.05, FDR‐corrected; Fig. 1B). Asymptomatic relatives also showed a reduction in PAF compared to healthy controls in 14 of the 17 parietal and occipital channels (*P* < 0.05, FDR‐corrected; Fig. 1B).

PAF remained significantly reduced compared to healthy controls in the sub‐group who were not on carbamazepine (Fig. S1), in the sub‐group of patients with poor seizure control (Fig. S2) and in both sub‐groups of unrelated patients and relatives (Fig. S3).

### EEG alpha correlates of BOLD‐fMRI network

**Figure 2:**
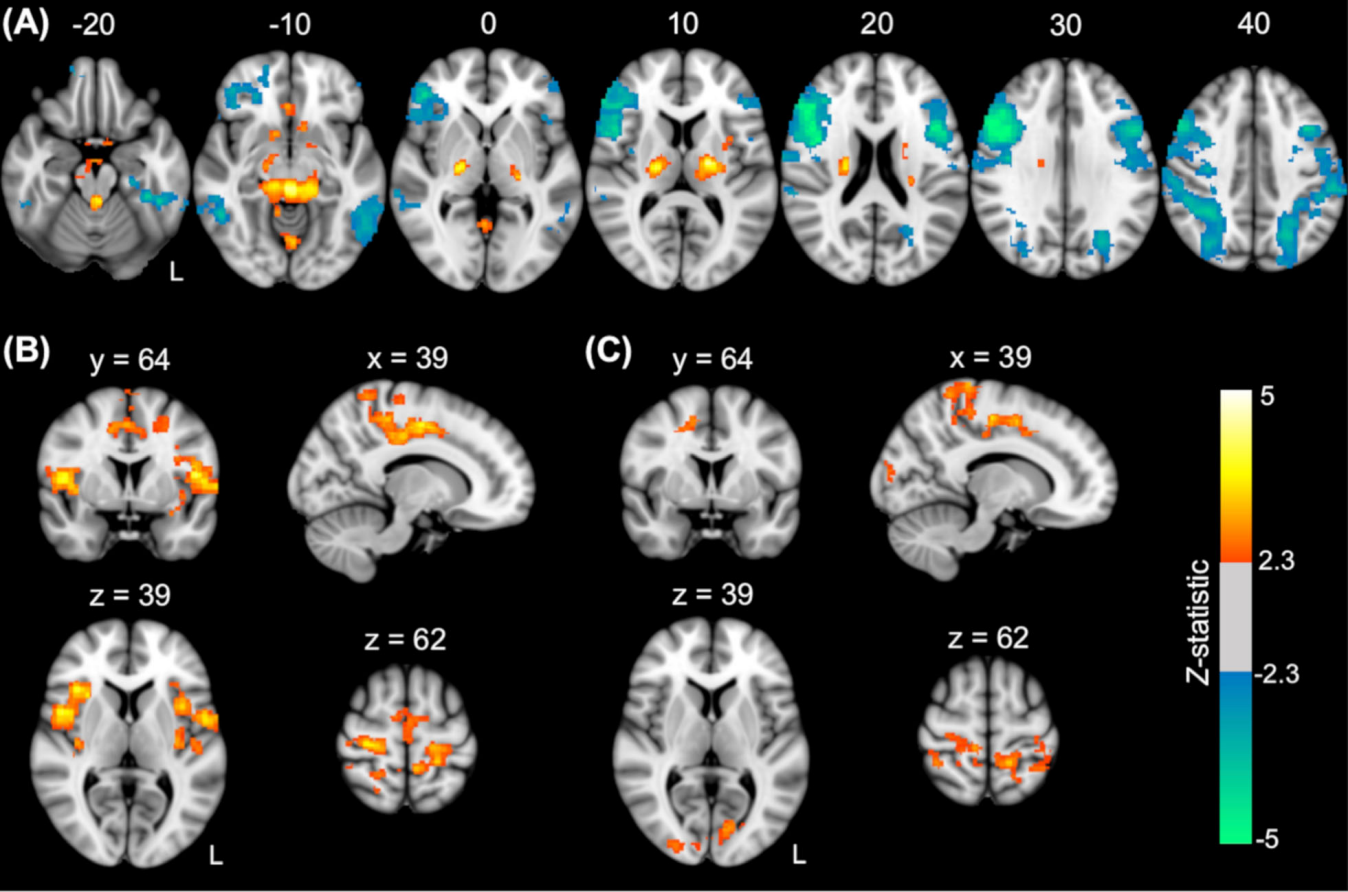
BOLD fMRI correlates of EEG alpha oscillations. (A) Regions showing positive and negative fMRI correlations with EEG alpha oscillations across all subjects. (B) Regions showing higher correlations with alpha oscillations in (B) patients and (C) asymptomatic relatives compared to healthy controls. Voxels in the sensorimotor region that were significantly different from controls in the relatives group had high overlap with voxels found significantly different from controls in the patient group. Images show *Z*‐statistics at a cluster threshold of *P* < 0.05 (FWE‐corrected). Positive values are shown in red/yellow and negative values in blue. MNI coordinates are shown above each slice. “L” represents the left or ipsilateral side. BOLD fMRI, blood‐oxygen‐level‐dependent fMRI; EEG, electroencephalography; MNI, Montreal Neurological Institute.

Regions showing positive and negative correlations of BOLD‐fMRI activity and EEG alpha oscillations across all subjects are shown in Figure 2A (*P* < 0.05, FWE‐corrected). Positive correlations were primarily found in the bilateral thalamus and parahippocampal gyrus, brainstem, and subcallosal cortex. Negative correlations were primarily seen in bilateral cortical regions in the dorsal attention network, including the middle and inferior frontal gyri, superior parietal lobes, lateral occipital cortices, and inferior frontal gyri.

Compared to healthy controls, patients showed significantly higher BOLD signal correlations with alpha activity in regions of the sensorimotor network, including the bilateral pre‐ and post‐central gyri extending into the supplementary motor area as well as in regions of the cingulo‐opercular/insular network, including the anterior cingulate, bilateral insulae and frontal and parietal opercula (*P* < 0.05, FWE‐corrected; Fig. 2B). Relatives also showed significantly higher BOLD signal correlations with alpha activity compared to healthy controls in regions of the sensorimotor network, including the bilateral pre‐ and post‐central gyri and anterior cingulate, in addition to the occipital cortex (*P* < 0.05, FWE‐corrected; Fig. 2C).

**Figure 3:**
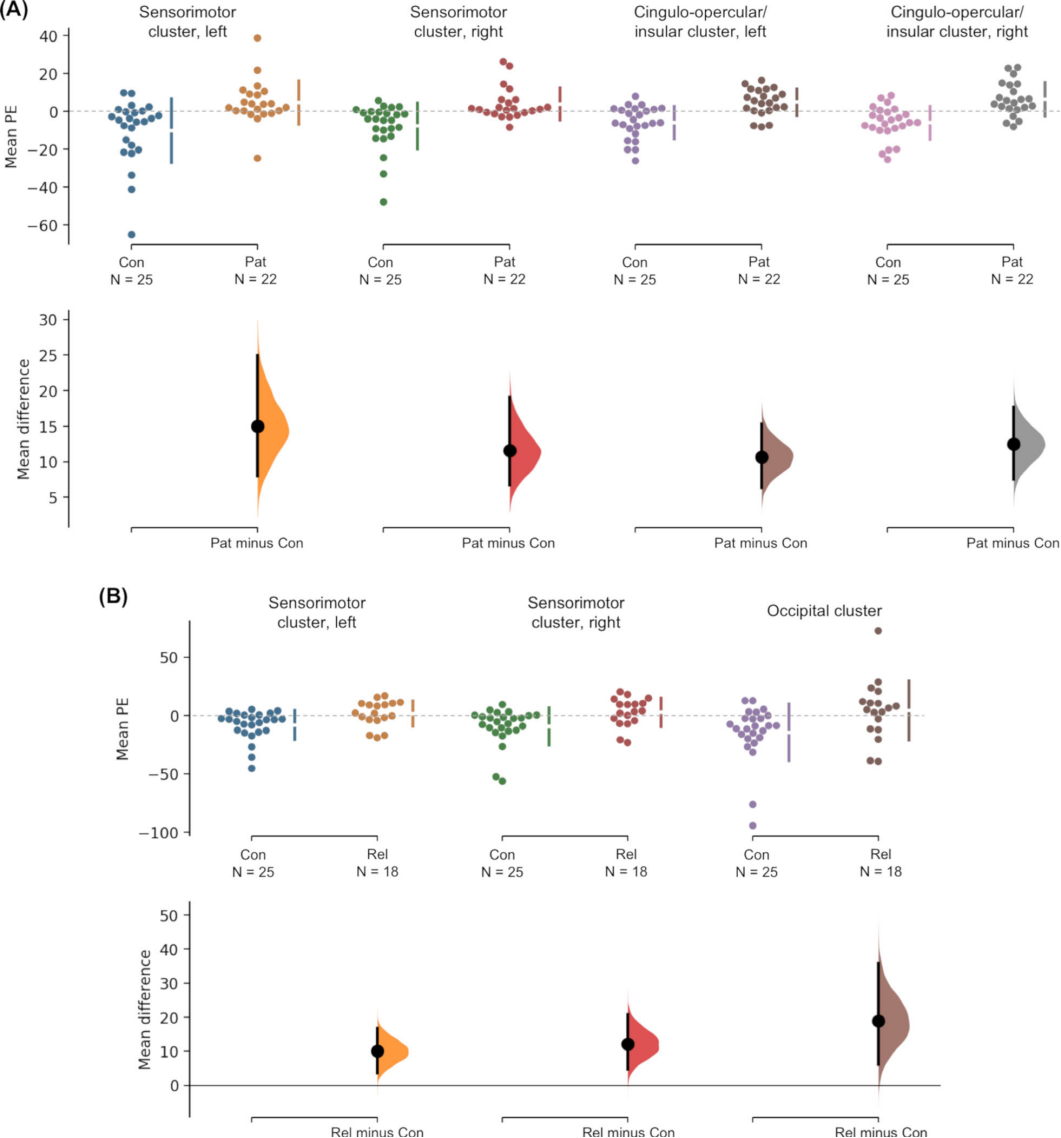
Mean group difference in correlation with alpha oscillation. (A) Clusters showing significantly higher correlation with alpha oscillation in patients compared to healthy controls. (B) Clusters showing significantly higher correlation with alpha oscillation in relatives compared to healthy controls. The mean parameter estimates are shown on the upper axes and the mean group differences shown on the lower axes as bootstrap sampling distributions. Mean differences are depicted as dots; 95% confidence intervals are indicated by the ends of the vertical error bars. Pat, patients with mTLE; Rel, asymptomatic relatives; Con, healthy controls. Figures created on http://www.estimationstats.com. mTLE, mesial temporal lobe epilepsy.

Both patients and relatives appeared to show a combination of higher activation (or higher correlation with alpha oscillations) and a failure of deactivation (i.e. a failure to decouple with alpha oscillations) compared to healthy controls who mainly showed reduced correlation with alpha in these regions (Fig. 3). There were no significantly lower BOLD correlations with alpha in either the patients or relatives compared to healthy controls.

The slope of the peak alpha power was not significantly different between groups: *F*(2, 62) = 1.361, *P* = 0.264, indicating that the level of vigilance between groups was not significantly different across the EEG‐fMRI recording.

## Discussion

The present study identified EEG PAF reductions and functional brain network alterations associated with alpha oscillations in both patients with mTLE and asymptomatic relatives compared to healthy controls. Analysis of the EEG power spectrum revealed a shift of the alpha rhythm toward lower frequencies in both patients with mTLE and asymptomatic relatives. The PAF shift was observed across all parietal and occipital EEG channels in patients, while in relatives it was seen in 14 of the 17 parietal and occipital channels. With simultaneous EEG‐fMRI, we showed that cortical regions in the sensorimotor network failed to deactivate in relation to alpha oscillations in both patients and asymptomatic relatives compared to healthy controls. In addition, patients also showed this pattern of increased fMRI activation and a failure to deactivate with alpha oscillations in the cingulo‐opercular/insular network. Reduced PAF and altered topographical distribution of EEG alpha power in patients with epilepsy have been reported previously, and we replicate this finding in independent data here. We show here, for the first time, that brain activity related to the alpha rhythm differs between patients with epilepsy and healthy controls. We also show, for the first time, that reduced PAF and altered brain activity related to the alpha rhythm, differ between asymptomatic relatives and healthy controls. These findings in relatives suggest that alterations in alpha activity in patients with epilepsy are not necessarily related to AEDs or seizures, and are an inherited endophenotypic predisposition to epilepsy, which is currently mechanistically unexplained.

### PAF shift

The shift in PAF toward lower frequencies in patients has been demonstrated previously in both patients with focal and generalized epilepsy, and is thought to be linked to poor seizure control.^28^ We also found evidence for a PAF decrease in asymptomatic relatives. Importantly, the asymptomatic relatives in this study were unmedicated, hence the reduced PAF cannot be attributed to the effects of antiepileptic drugs. The slowing of the alpha rhythm has also been reported in several other neurological and psychiatric disorders including depression and Alzheimer’s disease.^19^, ^44^ This suggests that the PAF on its own may not be a specific enough measure to serve as an endophenotype for mTLE and may instead point to a more general indicator of susceptibility to abnormal brain function.

### Brain network alterations related to alpha oscillations

Across the whole group of participants, we show positive correlations in the thalamus and negative correlations in the dorsal attention network. The pattern of positive and negative correlations of brain fMRI activity with alpha oscillations is largely in line with the literature in this field.^14^

Group comparisons showed a higher correlation of alpha oscillations with regions of the sensorimotor network in both patients with mTLE and asymptomatic relatives compared to healthy controls. In healthy controls, cortical regions mainly showed decreased activation with alpha oscillations. In patients, there was a combination of increased activation and a failure to deactivate in regions of the sensorimotor network and the cingulo‐opercular/insular network. Interestingly, in the sensorimotor network, relatives also showed the same pattern of increased brain activation and a failure to deactivate in relation to alpha oscillations.

Alpha oscillations are thought to govern cortical excitability, or a rhythmic inhibition, where increases in alpha power generally result in an increase in inhibition and hence a decrease in cortical excitability.^14^, ^45^, ^46^ The increased correlation between alpha oscillations and sensorimotor network activity observed in patients and relatives could suggest that alpha oscillations have a reduced effect of inhibition over sensorimotor network activity.

Compared to healthy controls, patients with mTLE also had higher correlations with alpha in the cingulo‐opercular/insular network. It is unclear what the link is between this network and alpha oscillations in mTLE. The cingulo‐opercular/insular network is thought to underpin “tonic alertness” through the modulation of alpha oscillations.^47^ The insular cortex has also been implicated in ictogenicity in mTLE.^48^, ^49^ As one of the few regions with direct connections to the cholinergic basal forebrain, it has been suggested that the link between the alpha rhythm and cholinergic basal forebrain activity is modulated by the insula.^50^

This study represents the first investigation of functional brain changes related to the altered alpha rhythm in patients with mTLE and asymptomatic relatives. The findings suggest that the shift in PAF and alterations in brain function related to the alpha rhythm may deserve further investigation as an endophenotype for mTLE and may not entirely be a consequence of anti‐epileptic drugs, seizures or HS.

## Author Contributions

MPR, GJB, and SNY conceived and designed the study. MK and RDCE contributed to primary patient referrals and clinical data. SNY, CT, and EA acquired the study data and conducted the analyses. SNY drafted the manuscript. All authors reviewed the paper.

## Conflicts of Interest

The authors report no conflicts of interest relevant to this work.

## Supporting information


**Figure S1.** EEG topographical plots for the alpha band in the sub‐group analysis investigating effect of carbamazepine therapy.
**Figure S2.** EEG topographical plots for the alpha band in the sub‐group analysis investigating effect of seizure control.
**Figure S3.** EEG topographical plots for the alpha band in the sub‐group analysis excluding the effect of relatedness.

**Table S1.** Further clinical details of patients in the study.

**Table S2.** Further details of relatives and clinical details of associated probands.
