# Supplementary material for: Heritability of alpha and sensorimotor network changes in temporal lobe epilepsy
Source: Ann Clin Transl Neurol. 2020 Apr 25;7(5):667–76. doi: 10.1002/acn3.51032 (PMC7261746; doi:10.1002/acn3.51032)

**Supplementary Figure 1. EEG topographical plots for the alpha band in the sub-group analysis investigating effect of carbamazepine therapy.** Group-averaged EEG topographical plots of (A) peak power and (B) peak frequency in the alpha frequency band. In the patients’ plots, channels that show a significant group difference from healthy controls are indicated by pink dots (p < 0.05, FDR-corrected across parietal and occipital channels only). “L” indicates the left or ipsilateral side. Pat (no CAR): patients with mTLE who are not taking carbamazepine, Pat (CAR): patients with mTLE who are taking carbamazepine, Con: healthy controls.


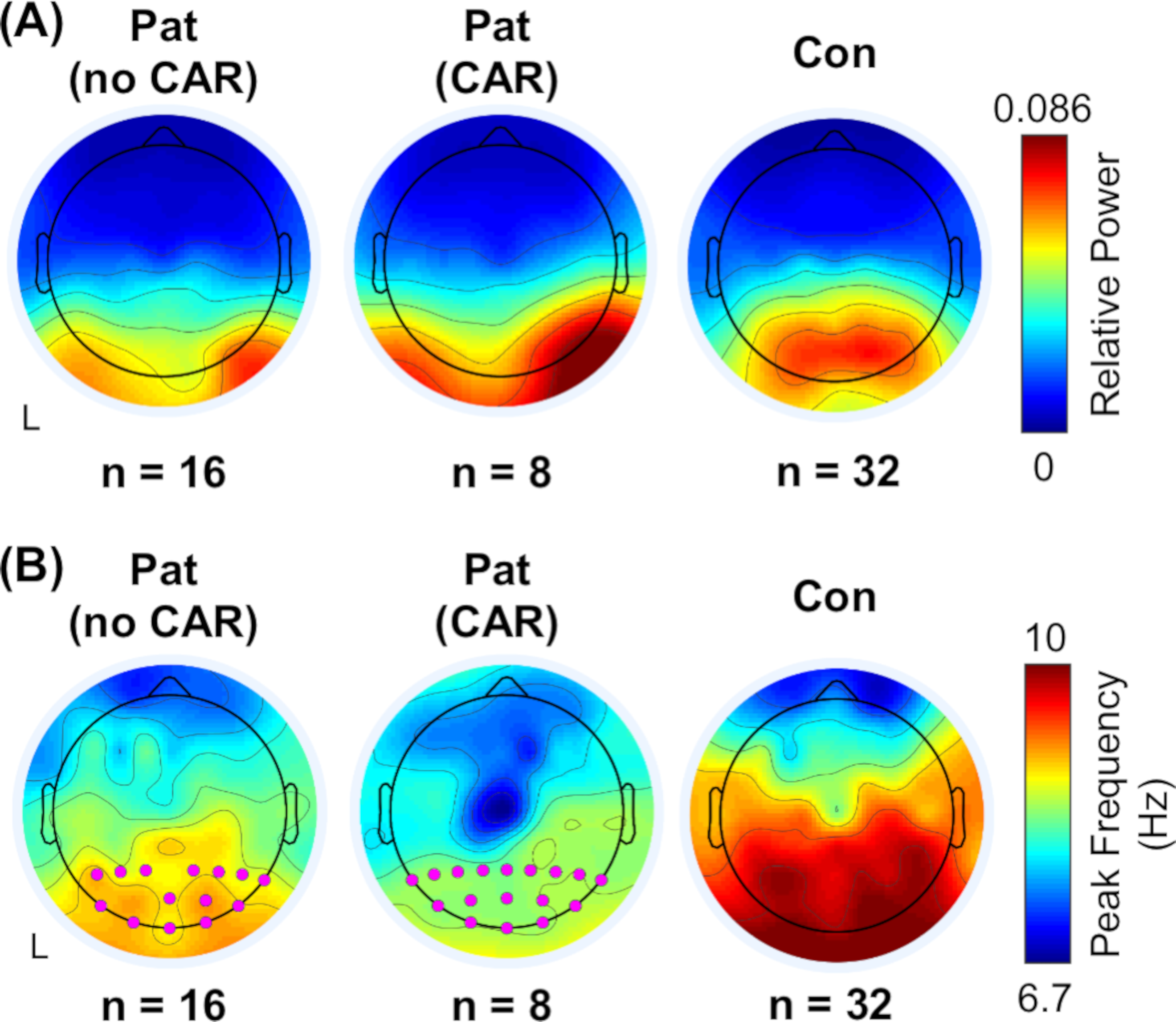


**Supplementary Figure 2. EEG topographical plots for the alpha band in the sub-group analysis investigating effect of seizure control.** Group-averaged EEG topographical plots of (A) peak power and (B) peak frequency in the alpha frequency band. In the patients’ plots, channels that show a significant group difference from healthy controls are indicated by pink dots (p < 0.05, FDR-corrected across parietal and occipital channels only). “L” indicates the left or ipsilateral side. Pat (poor control): patients with mTLE who have ≥ 4 seizure per year, Pat (good control): patients with mTLE who have < 4 seizures per year, Con: healthy controls.


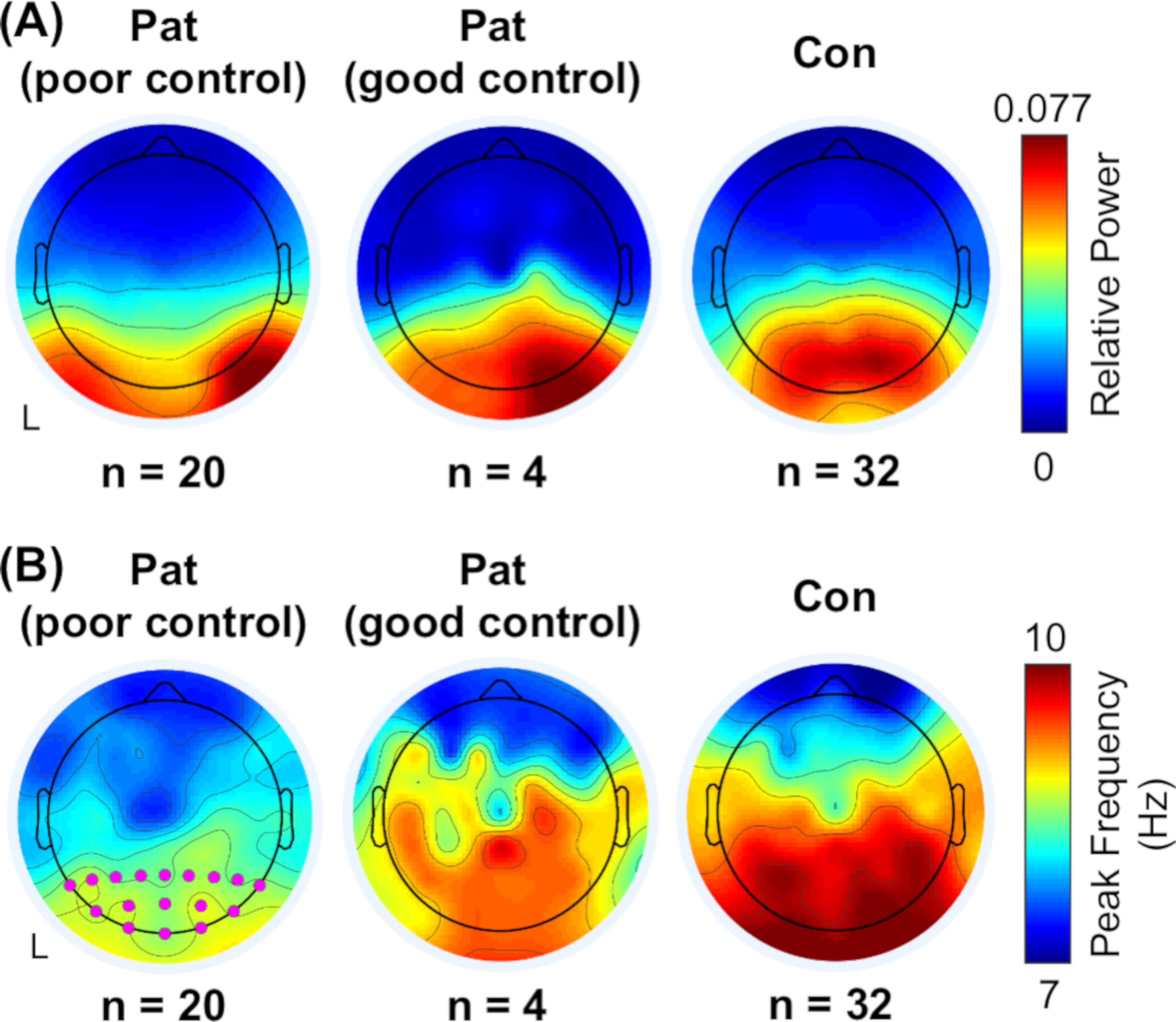


**Supplementary Figure 3. EEG topographical plots for the alpha band in the sub-group analysis excluding the effect of relatedness.** Group-averaged EEG topographical plots of (A) peak power and (B) peak frequency in the alpha frequency band. In the patients’ plots and relatives’ plots, channels that show a significant group difference from healthy controls are indicated by pink dots (p < 0.05, FDR-corrected across parietal and occipital channels only). “L” indicates the left or ipsilateral side. Pat: patients with mTLE (unrelated to relatives included in this analysis), Rel: asymptomatic relatives of patients with mTLE (unrelated to patients included in this analysis), Con: healthy controls.


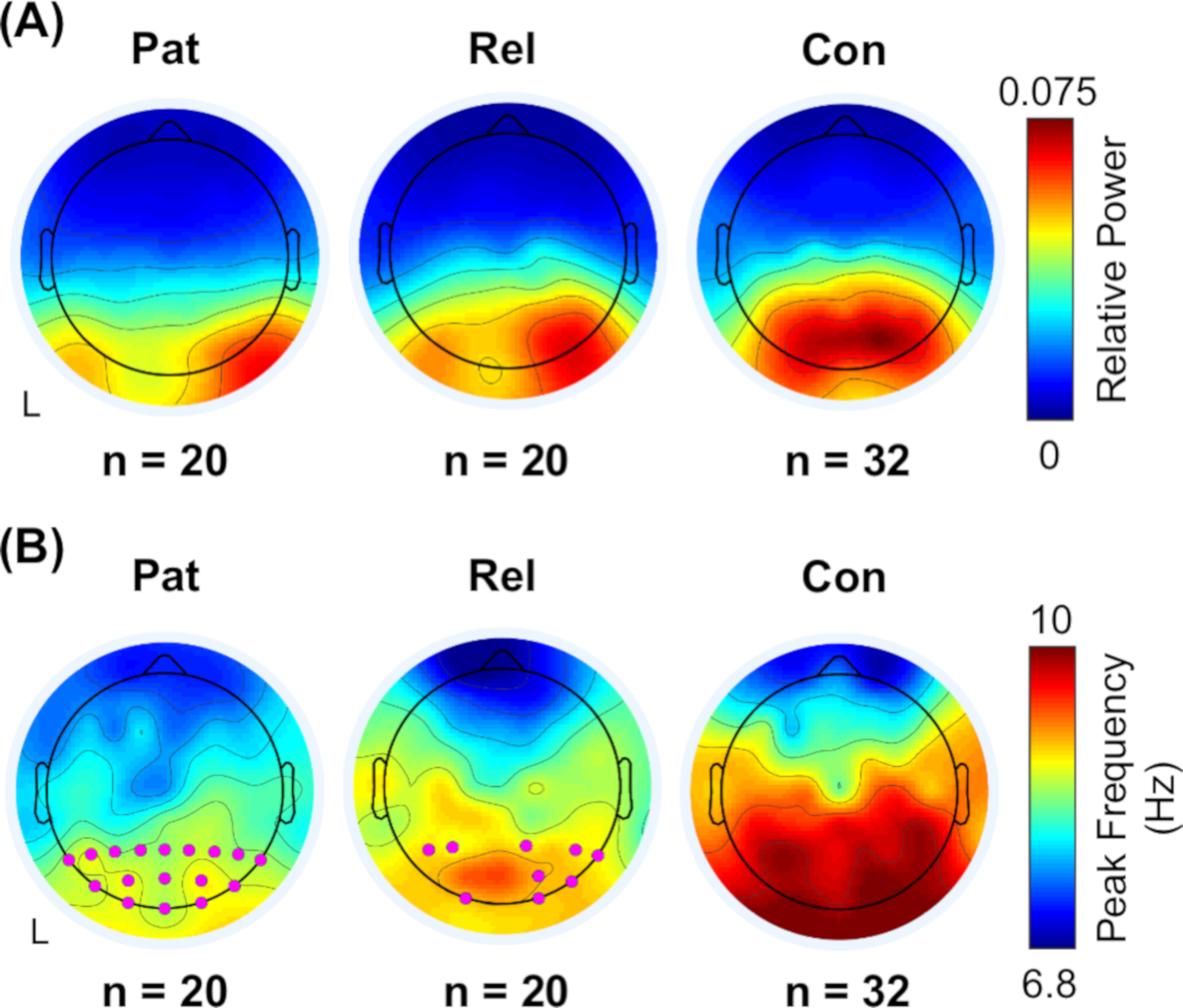

Supplement: Supplementary file 1 — Figure S1. EEG topographical plots for the alpha band in the sub‐group analysis investigating effect of carbamazepine therapy. Figure S2. EEG topographical plots for the alpha band in the sub‐group analysis investigating effect of seizure control. Figure S3. EEG topographical plots for the alpha band in the sub‐group analysis excluding the effect of relatedness. [file ACN3-7-667-s001.docx]
