# Supplementary material for: Heritability of alpha and sensorimotor network changes in temporal lobe epilepsy
Source: Ann Clin Transl Neurol. 2020 Apr 25;7(5):667–76. doi: 10.1002/acn3.51032 (PMC7261746; doi:10.1002/acn3.51032)
Supplement: Supplementary file 2 — Table S1. Further clinical details of patients in the study. [file ACN3-7-667-s002.docx]

**Supplementary Table 1.** Further clinical details of patients in the study.

| **ID** | **Age** | **Sex** | **Onset age** | **Localization features** | **Seizure frequency** | **Medication (daily dose)** | **Febrile seizures?** | **EEG?** | **EEG-fMRI?** |
| --- | --- | --- | --- | --- | --- | --- | --- | --- | --- |
| PAT01 | 41 | F | 24 | Right sided HS on MRI. | 9-10 | LMT (550); LEV (1500); PER (4); CLB (60) | * | Yes | Yes |
| PAT02 | 35 | M | 15 | Left sided HS on MRI. | 2 | LMT (400); VPA (900); CLB (10) | * | Yes | No |
| PAT03 | 43 | F | 20 | Left sided HS on MRI. Bitemporal seizure onset on intra-cranial EEG. | 3-7 | CAR (1400); LMT (200) | No | Yes | Yes |
| PAT04 | 57 | F | 5 | Left sided HS on MRI. Left temporal discharges on EEG. | 6-7 | LEV (875); CIT (30) | No | Yes | Yes |
| PAT05 | 22 | M | 16 | Left sided HS on MRI. | 20-24 | CAR (1200) | No | Yes | Yes |
| PAT06 | 34 | M | 11 | Right sided HS on MRI. Right anterior temporal spike and wave epileptiform discharges on EEG. | 2-3 | PHB (60); VPA (1200); OLA (7.5); CIT(*) | No | Yes | Yes |
| PAT07 | 52 | F | 15 | Loss of digitation in the left hippocampal head. Left temporal hypometabolism on FDG PET. Left temporal discharges on EEG. | 4 | LAB (150); CIT (50); LOR (2) | No | Yes | Yes |
| PAT08 | 51 | F | 31 | Left sided HS on MRI. | <1 | LAC (400) | * | Yes | Yes |
| PAT09 | 31 | M | 25 | Right sided HS on MRI. | 16-20 | CAR (800); LEV (200); CLB (10) | * | Yes | Yes |
| PAT10 | 48 | M | 33 | Right sided HS on MRI. | <1 | LEV (3000); TOP (100) | Yes | Yes | Yes |
| PAT11 | 31 | M | 21 | Right sided HS on MRI. | 3-4 | LEV (3000); ZON (200); CLN (2) | No | Yes | Yes |
| PAT12 | 58 | F | 47 | Left sided HS on MRI. | 3-4 | TOP (200); CLB (10) | * | Yes | Yes |
| PAT13 | 47 | M | 40 | Right sided HS on MRI | <1 | LEV (400) | * | Yes | No |
| PAT14 | 24 | M | 22 | Ectopic grey matter lateral to body of right hippocampus. Focal temporal lobe seizures and right temporal discharges on EEG. | 3-4 | CAR (400) | Uncertain | Yes | Yes |
| PAT15 | 25 | M | 23 | MRI normal. Left temporal focal seizures on EEG. Seizure semiology suggestive of left TLE onset. | 3-4 | VPA (800); TOP (300) | No | Yes | Yes |
| PAT16 | 43 | F | 40 | Left sided HS on MRI | 4 | CAR (800) | No | Yes | Yes |
| PAT17 | 23 | M | 22 | Right sided HS on MRI | <1 | ZON (200) | Yes | Yes | Yes |
| PAT18 | 47 | M | 15 | Normal MRI. Left temporal focal seizures on EEG. | 1-2 | CAR (600) | No | Yes | Yes |
| PAT19 | 24 | F | 22 | Left sided HS on MRI | * | * | No | No | Yes |
| PAT20 | 57 | M | 25 | Right sided HS on MRI | <1 | LMT (300) | * | Yes | Yes |
| PAT21 | 37 | F | 27 | Left sided HS on MRI | 12 | CAR (400) | Yes | Yes | Yes |
| PAT22 | 31 | F | 22 | Left sided HS on MRI | 3 | LEV (3000) | No | Yes | Yes |
| PAT23 | 44 | F | 1 | Left sided HS on MRI. | 4 | CAR (1200); CLB (10) | Uncertain | Yes | No |
| PAT24 | 22 | F | 18 | Left sided HS on MRI. Seizure semiology: posturing of right hand. | 1-2 | None at time of scan | No | Yes | Yes |
| PAT25 | 52 | M | 25 | Right sided HS on MRI. | 15 | LMT (750); PER (8) | * | Yes | Yes |

* indicates missing information; HS = Hippocampal Sclerosis; FS = Febrile Seizures. Seizure frequency is the approximate number of seizures per month.

Drug abbreviations: CAR = Carbamazepine, CIT = Citalopram, CLB = Clobazam, CLN = Clonazepam, LAC = Lacosamide, LEV = Levetiracetam, LMT = Lamotrigine, LOR = Lorazepam, OLA = Olanzapine, PER = Perampanel, PHB = Phenobarbitone, PHE = Phenytoin, TOP = Toparimate, VPA = Valproate, ZON = Zonisamide.
