# Supplementary material for: Heritability of alpha and sensorimotor network changes in temporal lobe epilepsy
Source: Ann Clin Transl Neurol. 2020 Apr 25;7(5):667–76. doi: 10.1002/acn3.51032 (PMC7261746; doi:10.1002/acn3.51032)
Supplement: Supplementary file 3 — Table S2. Further details of relatives and clinical details of associated probands. [file ACN3-7-667-s003.docx]

**Supplementary Table 2.** Further details of relatives and clinical details of associated probands.

| **ID** | **Age** | **Sex** | **Relationship to proband** | **Proband mTLE details** | **Proband in study** | **EEG?** | **EEG-fMRI?** |
| --- | --- | --- | --- | --- | --- | --- | --- |
| REL01 | 49 | M | Son of female patient | Age of onset 48. Left HS on MRI. Patient has well controlled seizures on medication and has not undergone surgery. | No | Yes | Yes |
| REL02 | 24 | F | Daughter of female patient | See Supplementary Table 1. | PAT07 | Yes | Yes |
| REL03 | 34 | M | Twin brother of male patient | See Supplementary Table 1. | PAT06 | Yes | Yes |
| REL04 | 37 | F | Daughter of female patient | See Supplementary Table 1. | PAT04 | Yes | No |
| REL05 | 60 | F | Mother of male patient | See Supplementary Table 1. | PAT11 | Yes | Yes |
| REL06 | 31 | F | Daughter of male patient | Age of onset 40. Right HS on MRI. No history of febrile convulsions. Patient has had right temporal lobectomy and amygdalohippocampectomy and is currently seizure free on medication. | No | Yes | Yes |
| REL07 | 17 | M | Son of female patient | Age of onset 10-15 years. Left HS on MRI. No history of febrile seizures. Patient has had amygdalohippocampectomy and was seizure free for 7 years, but seizures have recently recurred. Updated telemetry showed left sided abnormalities and independent right sided abnormalities, with some evidence for right sided hypometabolism on PET. | No | Yes | Yes |
| REL08 | 25 | M | Brother of female patient | Age of onset 19. Right HS on MRI. No febrile seizures. Patient has had right temporal hippocampectomy and was seizure free for a period before a recurrence of nocturnal seizures. Pathology confirmed hippocampal sclerosis. | No | Yes | Yes |
| REL09 | 30 | M | Son of female patient | Age of onset 10. Left HS on MRI. Patient has had left temporal hippocampectomy. Pathology confirmed hippocampal sclerosis plus FCD type 2b. Seizures remain post-surgery. | No | Yes | Yes |
| REL10 | 44 | M | Brother of male patient | Early age of onset. Right HS on MRI. Patient has not undergone surgery. | No | Yes | Yes |
| REL11 | 25 | M | Brother of male patient | See Supplementary Table 1. | PAT13 | Yes | Yes |
| REL12 | 54 | F | Mother of female patient | See Supplementary Table 1. | PAT19 | Yes | Yes |
| REL13 | 24 | F | Daughter of female patient | Early age of onset. Right HS on MRI. Patient has not undergone surgery. | No | Yes | Yes |
| REL14 | 18 | F | Sister of male patient | See Supplementary Table 1. | PAT15 | Yes | No |
| REL15 | 40 | F | Sister of female patient | Early age of onset. Bilateral HS on MRI. Patient has not undergone surgery. | No | Yes | Yes |
| REL16 | 40 | F | Sister of female patient | Early age of onset. Bilateral HS on MRI. Patient has not undergone surgery. | No | Yes | Yes |
| REL17 | 20 | F | Daughter of female patient | Age of onset 33. Left HS on MRI. Patient has had left temporal lobectomy. Pathology confirmed hippocampal sclerosis. Seizures remain post-surgery. | No | Yes | Yes |
| REL18 | 60 | F | Mother of female patient | Age of onset 5. Left HS on MRI showing volume loss and hyperintensity on T2-weighted MRI. Prolonged febrile seizures at 8 months. Patient has had left temporal lobectomy and is now seizure free. | No | Yes | Yes |
| REL19 | 28 | M | Brother of female patient | Age of onset 5. Left HS on MRI showing volume loss and hyperintensity on T2-weighted MRI. Prolonged febrile seizures at 8 months. Patient has had left temporal lobectomy and is now seizure free. | No | Yes | Yes |
| REL20 | 31 | F | Sister of female patient | Early age of onset. Right HS on MRI. Patient has not undergone surgery. | No | Yes | Yes |
| REL21 | 49 | M | Brother of female patient | Early age of onset. Left HS on MRI. Patient has not undergone surgery. | No | Yes | No |
| REL22 | 47 | M | Son of female patient | Early age of onset. Left HS on MRI. Patient has not undergone surgery. | No | Yes | No |
| REL23 | 44 | F | Daughter of male patient | Early age of onset. Right HS on MRI. Patient has not undergone surgery. | No | Yes | No |
